# Promoting a culture change in junior and youth sport in New Zealand

**DOI:** 10.3389/fspor.2022.811603

**Published:** 2022-09-12

**Authors:** Simon R. Walters, Vincent Minjares, Trish Bradbury, Patricia Lucas, Andrew Lenton, Kirsten Spencer, Simone Spencer

**Affiliations:** ^1^Sports Performance Research Institute, Auckland University of Technology, Auckland, New Zealand; ^2^North Harbour Basketball, Auckland, New Zealand; ^3^Massey Business School, Massey University, Auckland, New Zealand; ^4^Department of Sport, UNITEC, Auckland, New Zealand; ^5^Aktive, Auckland, New Zealand

**Keywords:** good sports, coach, parent, design-based research, transformative learning, children, behaviour change

## Abstract

This paper provides insight into the evolution of a project designed to address longstanding adult attitudes and behavioural issues in junior and youth sport in New Zealand. The project was funded by Sport New Zealand (Sport NZ) and implemented by Aktive, a charitable trust that works with national and regional partners to fund and deliver community sport in Auckland. Aktive collaborated with a team of junior and youth sport researchers, adopting a pragmatic, mixed methods design-based research (DBR) approach to co-design an educational delivery framework aimed at influencing attitudes and assumptions underpinning coaches, parents, and community sport leaders' behaviours. Transformative learning principles informed the delivery framework with the project reaching 4,222 participants. Research evaluations included multiple case studies, surveys, semi-structured interviews, and focus groups, which confirmed awareness of problematic beliefs. The programme expanded to Regional Sport Organisations (RSOs) and National Sport Organisations (NSOs) culminating in a nationwide rollout. The study highlights the effectiveness of theoretically informed adult behaviour change programmes in junior and youth sport, the benefits of programmes being underpinned by a rigorous pedagogical approach, and the benefits of sport organisations and researchers collaborating to design and deliver sustainable change initiatives that address belief systems underpinning current issues.

## Introduction and background

Sport has well-documented potential benefits for junior and youth participants, including increased physical activity levels, socialising opportunities, building leadership and teamwork skills, improved self-esteem, and having fun (Brenner, 2016). However, access to such benefits depends largely on the sporting environment created by adults (Rutten et al., 2011), who are influenced by prevailing dominant socio-cultural discourses that increasingly elevate the importance of winning in sport over development and enjoyable, ongoing participation (Walters et al., 2015).

The intensification of the junior and youth sport experience in New Zealand is not a recent phenomenon, rather it has evolved gradually over time. Since the 1950's, as in many other developed countries, children's leisure time has been increasingly spent in highly organised, regulated, adult-controlled sport and parents steadily devoted more time and resources towards supporting their children's participation (Ferkins et al., 2009a). As early as 1974, at the General Assembly of the International Federation of Sports Medicine in Melbourne “considerable concern about the strong emphasis that has come to be placed upon highly structured sports competition for young children” (Williams, 1974, p. 17) was noted. By the 1980's, evidence began to emerge that a significant number of New Zealand children were beginning to reject and withdraw from highly structured organised sports and in the early 1990's, concerns were being expressed about trends towards early specialisation and an increase in overuse injuries (Gerrard, 1993; Thomson, 2000).

Seemingly accelerating this intensification, a notion emerged that becoming an “expert” required 10,000 h of practice, which was further propagated through Malcolm Gladwell's 2008 book “Outliers: The story of success” (Gladwell, 2008). This theory, based on estimation, has been widely misused in sporting contexts (Ford et al., 2009), resulting in the belief that intensive, deliberate practice is the optimal pathway to expert or elite status. As an easily understood concept often cited in popular media, the 10,000-h “rule”' became a major driver of earlier intensive sport-specific training for children in western societies (McGowan et al., 2021).

The impact of the intensification of the sporting experience on young athletes is concerning. Recent New Zealand-based studies reinforced ongoing concerns expressed internationally in academic literature [for example, see Brenner (Brenner, 2016)], and by Sport New Zealand (Sport NZ), the government agency responsible for governing sport and recreation in New Zealand [for example, see Sport NZ (Sport NZ, 2015)], and the media [for example, see Howie (2013)]. These concerns relate to a junior and youth sport system focused on outcomes for the few rather than developmental opportunities for the many. This has created several issues that impact junior participants' enjoyment of sport through to more serious issues as young people transition to youth sport. These issues include: controlling adult sideline behaviour (Walters et al., 2012); early specialisation, increased training loads, and resultant overuse injuries (McGowan et al., 2019); coaches and players' problematic attitudes towards injuries (Whatman et al., 2018); attrition rates in youth sport (Walters et al., 2017); and controlling coach behaviours (McKenzie, 2013). At a time of concern over declining sport participation, Accident Compensation Corporation (ACC)^1^ statistics indicated a 60% increase in sport-related injuries for 10–14 year-olds over the past decade (Accident Compensation Corporation, 2019), with sport medicine professionals expressing concern over increased overuse injuries. Numerous investigations internationally and in New Zealand revealed many young people do not consider sport enjoyable [for example, see Walters et al. (2015), Brenner (2016)]. Consequently, sport leaders and junior and youth sport researchers in New Zealand have called for these concerns to be addressed (Johanssen, 2020).

In 2014, Sport NZ invited interested organisations to tender for the rights to develop a junior (ages 5–13) and youth (ages 14–18) sport “culture-change” programme. Aktive^2^ successfully tendered for the contract to design, implement, and evaluate the programme. The programme, subsequently named Good Sports, aimed to develop an adult education programme to initiate culture change and, ultimately, enhance junior and youth sport experiences by increasing adults' awareness of how their behaviour impacts this environment. This paper articulates the evolution of the Good Sports programme. In doing so, we draw upon a design-based research (DBR) framework to guide the process of design, implementation, and evaluation. Full ethical approval was granted for all aspects of this research through the primary author's university ethics committee and all participants provided fully informed assent/consent.

## Methodology

This study drew upon our expertise in junior and youth sport research, while being situated within a “real world” context where the translation of knowledge into “on the ground” practice and impact was imperative to the programme's success. As highlighted by Holt et al. (2018), despite extensive junior and youth sport research in a number of domains there is minimal evidence of adequate translation of knowledge into practice. Therefore, this study was situated within a pragmatism paradigm, which is the subject of considerable debate [for further detail see Simpson (2018)]. Pragmatism has been defined as a “philosophy that attends to the practical nature of reality” (Shaw et al., 2010, p. 514) with considerable potential to inform aspects of research “that are concerned with the dynamics of human and social practice” (Simpson, 2018, p. 54). Pragmatism has gained recognition as a paradigm by a number of researchers [e.g., see Creswell and Plano Clark (2007)] and is increasingly seen in studies that include a range of methods to achieve meaningful results to inform policy and practice (Creswell and Plano Clark, 2007; Shaw et al., 2010). Adopting a pragmatic stance enables researchers to utilise the most appropriate methods to address the research questions (Shaw et al., 2010). This study adopted a DBR approach utilising mixed methods, bringing researchers and industry practitioners together, with the aim of developing a programme that influences adult attitudes and beliefs. In line with Shaw et al. (2010) we argue that mixed methods research conducted within a pragmatist view can address the “multiple practical challenges faced by practitioners better than either qualitative or quantitative research approaches in isolation” (Shaw et al., 2010, p. 510).

### Design-based research

Adopted relatively recently as an approach in educational research, a key aspect of DBR is the simultaneous design of education initiatives and evaluation of their effectiveness in real-world settings (Walker and Kettler, 2020). DBR comprises eight main characteristics that provided a framework for the present study: (1) being situated in real educational contexts; (2) focusing on designing and testing significant interventions; (3) using mixed methods; (4) involving multiple iterations; (5) incorporating collaborative partnerships between researchers and practitioners; (6) containing evolution of design principles; (7) including comparison to action research; and (8) having practical impact on practice (Design-Based Research Collective, 2003; Anderson and Shattuck, 2012).

DBR strengthens the impact, transfer, and translation of educational research into improved practice (Anderson and Shattuck, 2012) to develop domain-specific learning theories and processes that support learning. Brown (1992) pioneered DBR to link laboratory studies of learning with studies of complex instructional interventions. Brown's approach was designed to enable researchers to systematically alter elements of the design context. Each alteration becomes an experiment allowing theory to be generated and examined in naturalistic contexts.

DBR, therefore, enables complex educational problems requiring holistic examination to be addressed (Plomp, 2013), and addressing adult behavioural issues in junior and youth sport is multi-faceted and complex. Adult attitudes and beliefs towards junior and youth sport are often deeply entrenched, emanating from multiple influences, including culture (Bhalla and Weiss, 2010), gender (Coakley, 2006), class differences and generational changes (Stefansen et al., 2018), and a prevailing “win-at-all-costs” societal discourse (Walters et al., 2015).

In line with a DBR approach, Good Sports evolved as a collaboration between industry and academia. Aktive managed the project, co-designing with local junior and youth sport researchers (Aktive, 2019). In 2014, the project manager and steering group composition was confirmed. Two academics sat on the steering group and their initial role was to lead the monitoring and evaluation of the project. However, the steering group felt that creating a wider research group would ensure resources developed were underpinned by recent relevant literature, whilst simultaneously enabling researchers to collaborate in the design. A research group, composed of six academics from three tertiary institutions, and three postgraduate and three undergraduate students, was established. Steering group and research group meetings were held monthly with regular informal meetings between the research group leader and project manager.

**Figure 1:**
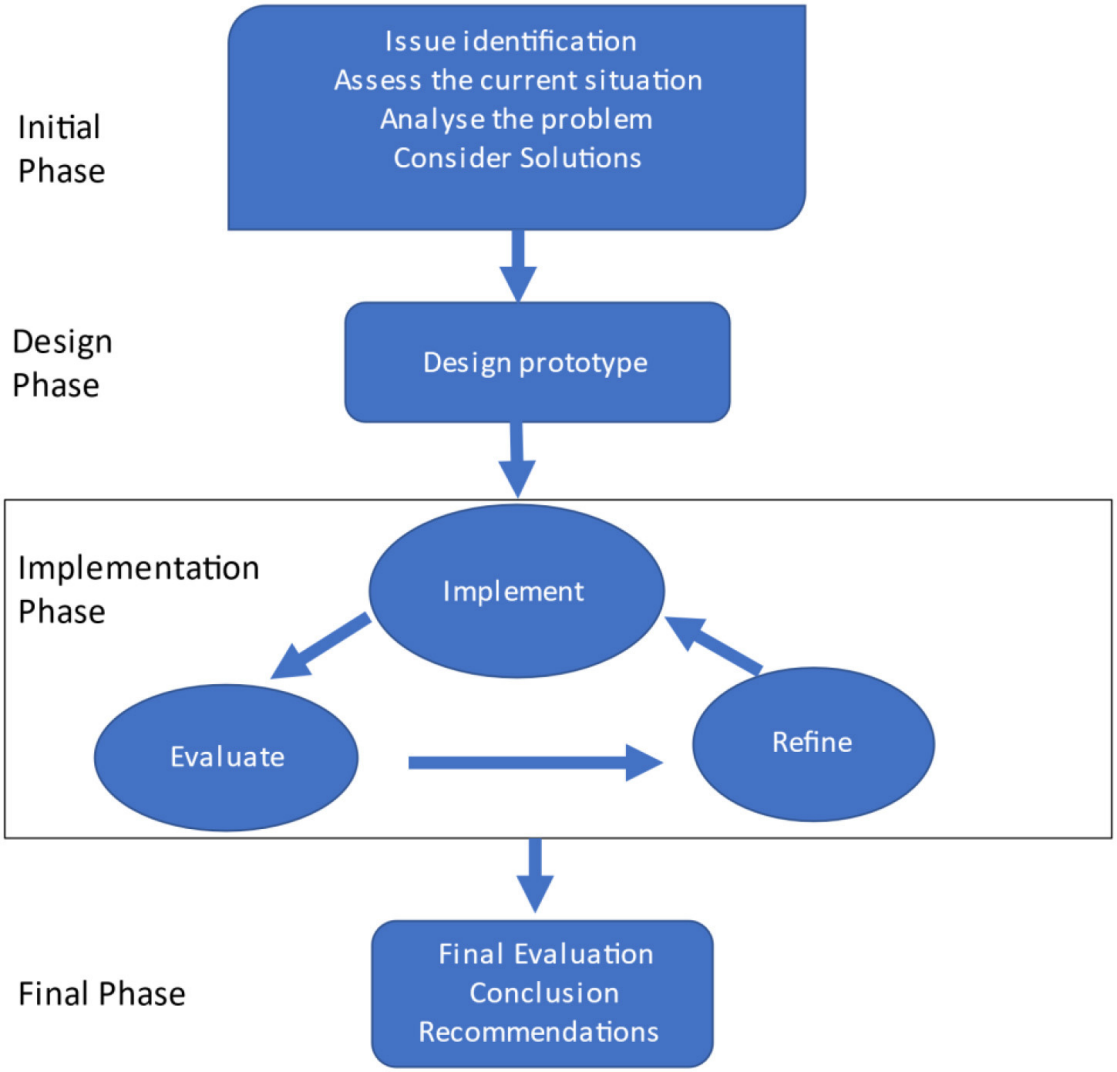
Design-based research model [adapted from Ferkins et al. (2009b), Jen et al. (2015)].

Two key aspects of DBR include evolving design principles, and a process comparable to action research. Following a framework that guides the writing of action research projects, reports typically take a narrative form (McNiff, 2013). In this paper, the story of Good Sports' evolution is told. In doing so, the narrative is presented in four key phases (see Figure 1).

Results from DBR studies can help researchers and practitioners develop in-depth understanding of how interventions work (or not) in specific contextual settings (Jen et al., 2015). The search for generalisable knowledge has been the dominant approach in social science research; however, in this study we aim to provide opportunities for “naturalistic generalization” (Stake, 2006), whereby readers can relate similarities and differences from the research to their own experiences.

### The initial phase

#### Issue identification and assessing the current situation

As detailed in the introduction and background section, junior and youth sport issues were clear. New Zealand-based evidence supports a range of international evidence suggesting an overly competitive and controlling junior and youth sporting environment impacted participant enjoyment across all ages and influenced dropout rates for young people moving into their early teenage years [for example, see Kidman et al. (1999), Walters et al. (2011, 2015, 2017), McKenzie (2013)]. During the initial phase, the research group focused on identifying some of the key international initiatives introduced to address junior and youth sport issues that could, potentially, be adapted within the New Zealand setting.

A research assistant was employed and tasked with locating and collating peer reviewed literature that addressed two key initial questions: What evidence exists of initiatives and/or interventions that have successfully generated shifts in adult attitudes and beliefs towards the role and purpose of sport for young people? Of these, how many have been scaled to a national level? The findings were then discussed by the research team in conjunction with the steering group. Of the interventions located for discussion, the research team's main interest was understanding what worked and why and whether any of these programmes could be adopted in New Zealand. Of the programmes located, the discussion narrowed to consider national regulatory approaches, the English Football Association's (FA) Respect programme in England, the Positive Coaching Alliance (PCA) and Positive Coaching Scotland (PCS) programmes in the USA and Scotland, respectively, and coach and parent education programmes.

### National regulatory approaches

Norway was the first country to formally regulate children's rights and activities in sport (through Children's Rights in Sport (CRS) and Provisions on Children's Sport), with the CRS declaration underpinning its junior and youth sport system. National Sport Federations adopted CRS, with financial penalties for failure to comply (Ellingsen and Danielsen, 2017). The United Nations Convention on the Rights of the Child (UNCROC), explicitly included in Swedish sport policy since 2009, was incorporated by law into Swedish sport on January 1, 2020. Puerto Rico also introduced law supporting policy development for protecting minors in sport (Farrey, 2020). However, the effectiveness of these approaches in changing children's organised sport experiences is uncertain. Eliasson (2017) determined Swedish athletes and adult coaches were unaware of UNCROC's impact on children's rights in sport policy, and in Norway CRS has not been widely embraced due to its perceived negative impact on achievement and short-term sporting success:

[In] the public debate in Norway, the CRS seem at times to be viewed as a hindrance to bring about top-level achievement in sports [...] and it seems to be less focused upon the children's rights perspective and the best interests of the child (Ellingsen and Danielsen, 2017, p. 416).

However, Ellingsen and Danielsen found coaches' attitudes typically aligned with children's best interests, with some exceptions, such as in gymnastics.

Despite these policies being introduced, however, it should be noted that the issue of dropout in sport was not as evident in Nordic and Scandinavian countries (for example Denmark, Sweden, Norway, Finland and Iceland) (Green et al., 2015). In Norway, at the same time as countries like New Zealand were witnessing a decline in youth sport participation, sporting participation trends were on the increase, especially for 16–19 year-olds. Social (e.g., parental support), cultural (e.g., the perceived role of sport, and the growth of “lifestyle” sports), and socio-economic conditions were deemed important factors in explaining the increasing sport participation in Norway. Thus, Green et al. argue, countries like Norway are not true comparators for others, such as the UK (and New Zealand), and “it is likely to be the greater socio-economic equalities in Scandinavian countries such as Norway that make them unrealistic benchmarks for sports participation elsewhere” (Green et al., 2015, p. 285)]. In addition, research team discussions concluded that legislative and policy changes at the levels witnessed in Scandinavia were beyond the scope of this pilot Good Sports project.

### National initiatives

In 2008, the FA implemented “Respect,” a “prosocial behaviour change programme” (Brackenridge et al., 2011, p. 175), aimed at reducing abusive sideline behaviour towards junior and youth football participants and referees in England (Brackenridge et al., 2011). Respect included a public information campaign, with policy decisions made by senior management, and subsequent programmes assigned to a wider community workforce for compliance (Cleland et al., 2015; Webb et al., 2017). Despite short-term benefits (enhanced player and referee enjoyment and improved behaviour), game-day interventions' (designated spectator areas, codes of conduct with sanctions, and only captains allowed to talk to referees) long-term results were perceived to be ineffective as referees still experienced abuse (Cleland et al., 2015, 2018).

“Play by the Rules,” promoting integrity in a broad range or junior and youth sport issues in Australia was initially led by an Australian regional government agency. The programme evolved into a multi-agency national collaboration promoting integrity in a broad range of junior and youth sport issues providing online training and resources (Play by the Rules, 2019). However, Kerr et al. (2014) disputed whether its objectives (to enhance awareness and knowledge) were achieved and found that most child-athlete protection initiatives they examined were not underpinned by theory and research.

### Coach and parent education programmes

Internationally, coaches and parents' influence on junior and youth sport experiences has drawn considerable attention (Keegan et al., 2008; Gearity and Murray, 2010). Table 1 outlines three sample initiatives.

**Table 1:** International coach and parent education programmes.

**Programme**	**Author(s)**	**Emphasis/goals**	**Methods**	**Adaptations**	**Country**	**Benefits**
Coach Effectiveness Training programme (CET)	Smith and Smoll (1997) and Topor and Gill (2008)	Autonomy-supportive environments	Behaviour change workshops delivered to thousands of junior and youth sport coaches	Mastery Approach to Coaching (MAC) (Smith et al., 2007; Smoll et al., 2007)	United States; Canada; Israel	Increased task goal orientations; decreased ego goal orientations; reduced sport anxiety (Smith et al., 2007; Smoll et al., 2007); greater enjoyment; feel more positively towards coaches/teammates (Keegan et al., 2008)
Positive Coaching Alliance (PCA)	Fry et al. (2020, p. 6)	“Transform the culture of youth sports away from an entertainment culture to a Development Zone™ culture” to address low junior and youth sport participation rates in the United States resulting from increasing negative experiences and “win-at-all-costs” mentalities	Advisory board structure, partnerships and media campaigns with elite coaches, athletes, sport leaders, and researchers. Workshops, programmes, and online resource platform for junior and youth sport stakeholders	Positive Coaching Scotland (Allen and Cronin, 2016; Scottish Football Association, 2021)	United States; Scotland	Workshops support coach-oriented character development, fostering positive junior and youth sport connections (Ferris et al., 2015)
Respect in Sport (RiS)		Prevent abuse, harassment, and discrimination in junior and youth sport	Developed an in-class curriculum and e-learning platform	Parent Program, a subset of RiS, aimed at educating parents	Canada	Reduction in participants' antisocial behaviour towards opponents and improved personal and social skills (Tamminen et al., 2020)

As outlined in Table 1, these initiatives were underpinned by research, and their reported success was supported by a face-to-face workshop approach.

#### Assessing the problem and considering solutions

These worldwide initiatives symbolised increasing concern for junior and youth sport participants' welfare. Evidence suggests some success for coach and parent education programmes informed by research directly related to junior and youth sport experiences. For example, RiS and PCA developed practical tools, derived from extensive sport psychology research (Fry and Moore, 2019), that sport leaders, coaches, parents, and athletes used to enhance junior and youth sport experiences and achieve positive holistic outcomes (Fry et al., 2020). Consequently, RiS appeared to be more effective than Respect at creating positive junior and youth sport environments (Nirmal, 2010; Tamminen and McEwen, 2015).

**Figure 2:**
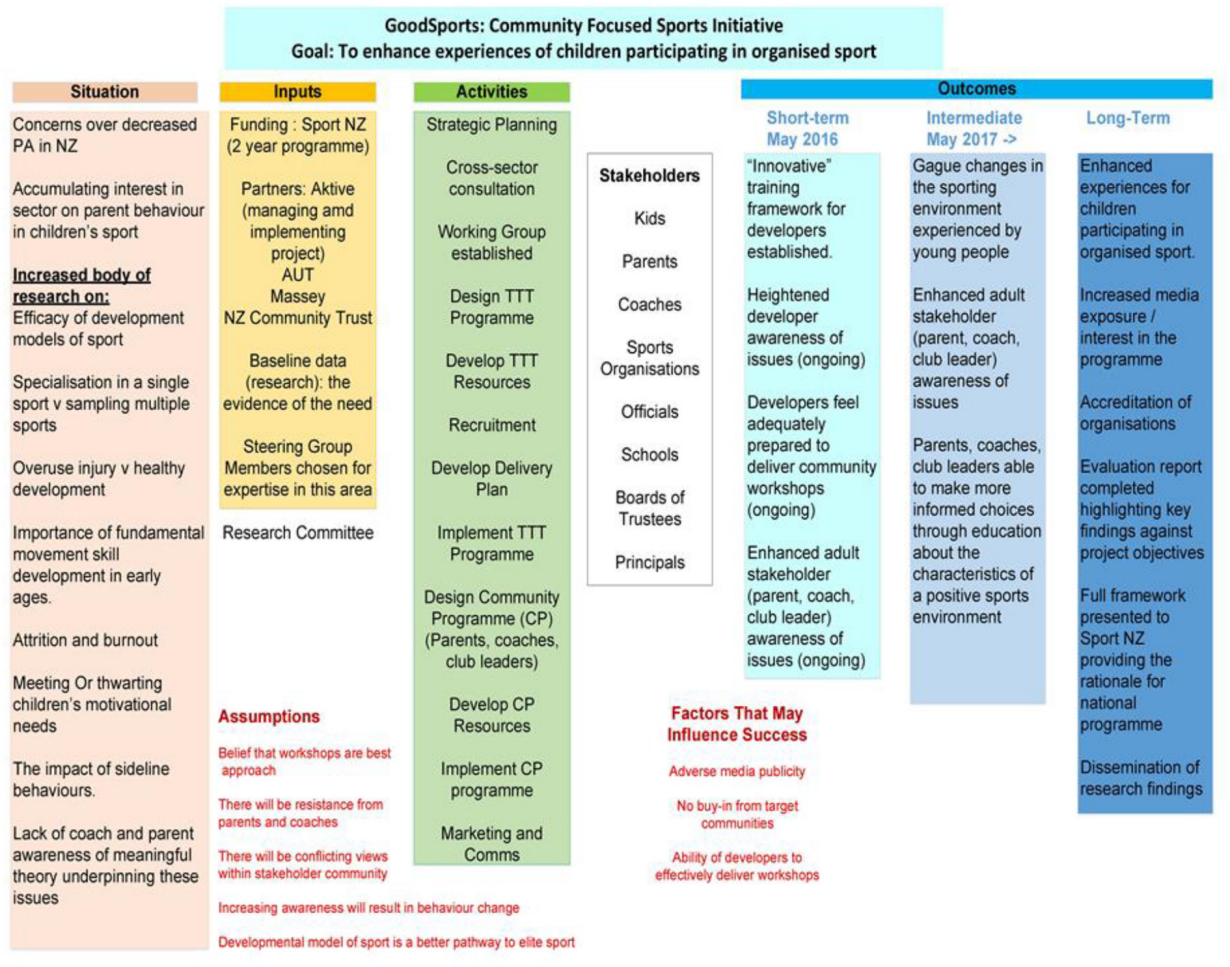
Programme logic model. TTT, Train the Trainer.

At this stage, a programme logic model was developed to help map out the findings of our analysis, determine the programme's short- and long-term aims, and inform the development of a solution (see Figure 2). From the research team's perspective, adult behaviour change programmes theoretically grounded in research were needed. The steering group and project team agreed that collaboration between industry practitioners and academic researchers could effectively advance knowledge by integrating more systematic approaches to programme development.

In considering solutions, there was substantial support for in-person workshop-based programmes, underpinned by robust research, aimed at influencing adult attitudes, beliefs and, ultimately, behaviour. PCA and PCS were deemed appropriate to Good Sports and adopting them in New Zealand was considered. However, high cost associated with implementing these programmes in New Zealand and lack of cultural specificity made them unsuitable. Instead, the Good Sports team developed a programme underpinned by a rigorous pedagogical approach, using New Zealand-based academic expertise to design its teaching and learning framework and inform resource design. Thus, developing a theoretically underpinned adult education delivery model, with trained facilitators using in-person workshops targeting belief systems underpinning adult behaviour in junior and youth sport, was decided upon.

Ultimately, the primary aim of this study was to evaluate the way and degree to which the Good Sports programme influenced the attitudes and assumptions of programme participants and key stakeholders (Figure 1). The research had three key objectives. First, to assess the effectiveness of the project in promoting awareness and understanding of the benefits of development-oriented practices in junior and youth sports. This included how effective the programme was influencing coaches, parents and sport leaders' perceptions that the purpose of sport is positive development, and the role of adults facilitating development through positive, non-controlling and long-term focused behaviours. Specifically, this project assessed Good Sports' influence on sport leaders' perceptions systems and structures that could be improved to facilitate youth development. Second, to assess the efficacy of a train-the-trainer, workshop-based model for national scalability. Third, to provide a framework that the national agency, Sport NZ, could leverage for nationwide programming.

### The design phase

The review of international programmes and subsequent team discussions indicated improved understanding of *how* education efforts transfer to applied settings was required (Gould, 2016). Guided by an approach used previously by University of Sterling researchers where evaluation research team members were embedded within the everyday practices of PCS (Lynn et al., 2013), an inside researcher (IR [second author]) was embedded in all aspects of our project, including as a “lead trainer,” informing the educational framework's design and facilitating modules for multiple stakeholder groups.

DBR “is interventionist by nature” (Walker and Kettler, 2020, p. 26) and the purpose of the project was to design and evaluate a programme sport organisations nationwide could use for effective junior and youth sport adult education delivery. It included the following aims:

a. evaluate the effectiveness of researchers working alongside industry partners to co-design a theoretically informed adult behaviour change programme;
b. promote adult attitudes and behaviours that enhance junior and youth sport experiences;
c. enable knowledge transfer to real-world settings;
d. investigate and evaluate multiple stakeholders' experiences of the programme;
e. make recommendations to Sport NZ for developing sustainable approaches to adult behaviour change in junior and youth sport.

The research group's objectives focused on four main focus areas: developing a pedagogical framework to underpin face-to-face workshop conversations; developing resources that would effectively support workshop delivery; developing the Good Sports programme infrastructure; and programme monitoring and evaluation.

#### Transformative learning: A pedagogical framework

Good Sports' educational aims were complex; therefore, a transformative learning pedagogical foundation (Mezirow, 2011; Sterling, 2011; Taylor, 2011) was utilised to engage participants. Transformative learning is a reconstructive theory that “provides a model for understanding how adults learn in various cultural settings” (Mezirow, 2011, p. 21) and offers a mechanism for understanding and fostering change in adult meaning-making systems (Kroth and Cranton, 2014). Specifically, it is a process resulting in transformation of “problematic frames of reference to make them more inclusive, discriminating, reflective, open, and emotionally able to change” (Mezirow, 2011, p. 22) Studies based on transformative learning focus on complex concepts demanding paradigm change, such as environmental sustainability (Sterling, 2011), and veganism (Lestar, 2020). It embraces Habermas's (1984) distinction between instrumental and communicative learning (Mezirow, 2011). Communicative learning uses discourse to interrogate inherent relationships between beliefs, their underpinning assumptions, and expression through language and action. Drawing upon Mezirow (2000) work, Taylor (2011) summarised how transformative learning unfolds:

It entails the identification of problematic ideas, beliefs, values, and feelings; critically assessing their underlying assumptions; testing their justification through rational discourse; and striving for decisions through consensus building (p. 3).

Bateson (1972) seminal essays on the ecology of mind provided philosophical grounding. Bateson's model distinguishes the depth of individual learning based on the extent that learning experiences challenge an individual's most basic assumptions. Whereas, first-order learning does not fundamentally alter an individual's assumptions and/or beliefs, second- and third-order learning “touches our deeper levels of knowing and meaning, and, by doing so, then influences our more immediate and concrete levels of knowing, perception, and action” (Sterling, 2011, p. 22). Bateson's distinction between orders of learning reinforces a key design element in Good Sports: the use of in-person workshops that trigger critical reflection in adults. According to Sterling (Sterling, 2011), second-order changes do not inherently occur as functions of everyday experiences, but through events that stimulate contradictions leading to critical reflection upon previously unexamined assumptions. On that basis, Good Sports sought to establish conditions that would trigger reflection on beliefs.

Good Sports workshop design was based on Taylor's (2011) six elements of transformative learning. First, Taylor identified prior individual experiences as the starting point for personal transformation. Powerful stories and scenarios provided workshop course content to trigger emotional responses from participants. Mezirow interpreted such triggers as “disorienting dilemmas”, intense experiential activities that can act as catalysts for critical reflection on “the integrity of deeply held assumptions and beliefs based on prior experience” (Taylor, 2011, p. 7). Second, critical reflection promotes transformational learning by supporting participants' awareness of conflicting thoughts, feelings, and actions triggered by disorienting dilemmas. On this basis, open-ended questions and writing activities intentionally followed the introduction of powerful stories. Third, dialogue, a medium for transformation to occur. Specifically, trustful communication, as opposed to point-counterpoint analysis, provides validation for participants to make sense of critical reflection. Requisites for trustful communication include freedom from coercion, openness to alternative points of view, showing empathy, equal opportunities to participate, and encouragement to seek understanding. Fourth, Taylor (2011) proposed that efforts to intentionally facilitate transformative learning should adopt a holistic orientation, due to its inherently emotional nature. Efforts to build community, establish rituals, and demonstrate empathy reinforce this. Facilitators support holistic orientation by sharing their vulnerabilities and encouraging active listening. Fifth, transformative learning demands awareness of context, whereby effort is required to express appreciation and awareness of learners' unique personal situations, including differences in readiness for change and/or interpretation of content. Facilitators display appreciation by embracing diverse responses to open-ended questions that encourage learners to make sense of issues and concepts related to their own situations. Finally, authentic relationships, fostered by facilitators making time for informal interactions, involved encouraging learners to share stories and implementing one-on-one interactions into the session's design.

#### Resource development: The good sports spine and delivery framework

**Figure 3:**
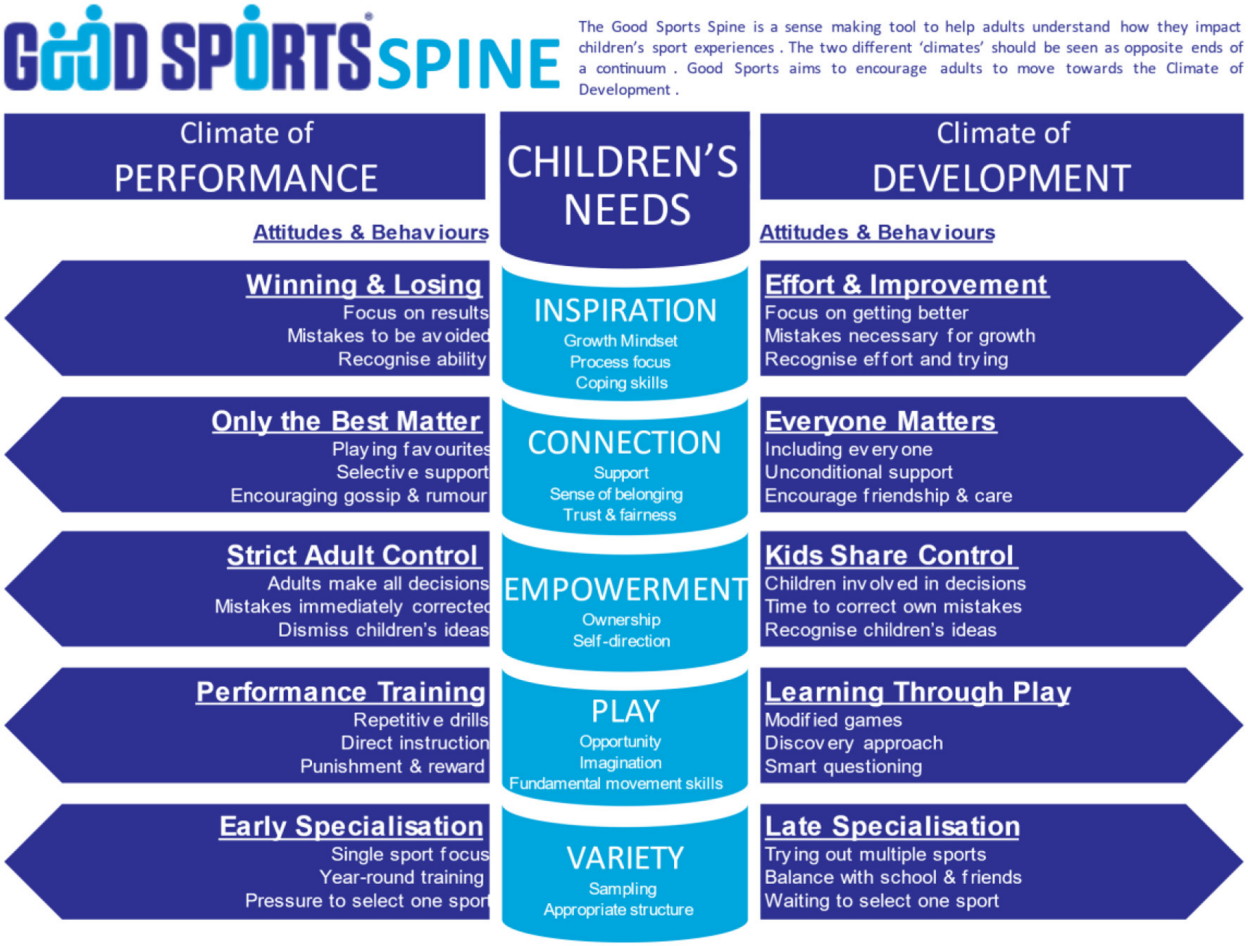
The Good Sports spine (Aktive, 2019).

The Good Sports Spine (Figure 3) was co-designed by the IR and Aktive team to develop a landscape of beliefs and establish direction of change for learners. Based on self-determination theory (SDT) (Deci and Ryan, 2000, 2002), growth mindset (Dweck, 2012), and the Developmental Model of Sport Participation (DMSP) (Côté et al., 2012), the spine provided an evidence-based tool that enhanced the transformative potential of critical reflection and discussion during workshops. The spine distinguished between two “climates”: (1) performance and (2) development, resting on the assumption that children's needs are best satisfied when adults' attitudes and behaviours align with a “climate of development.”

**Figure 4:**
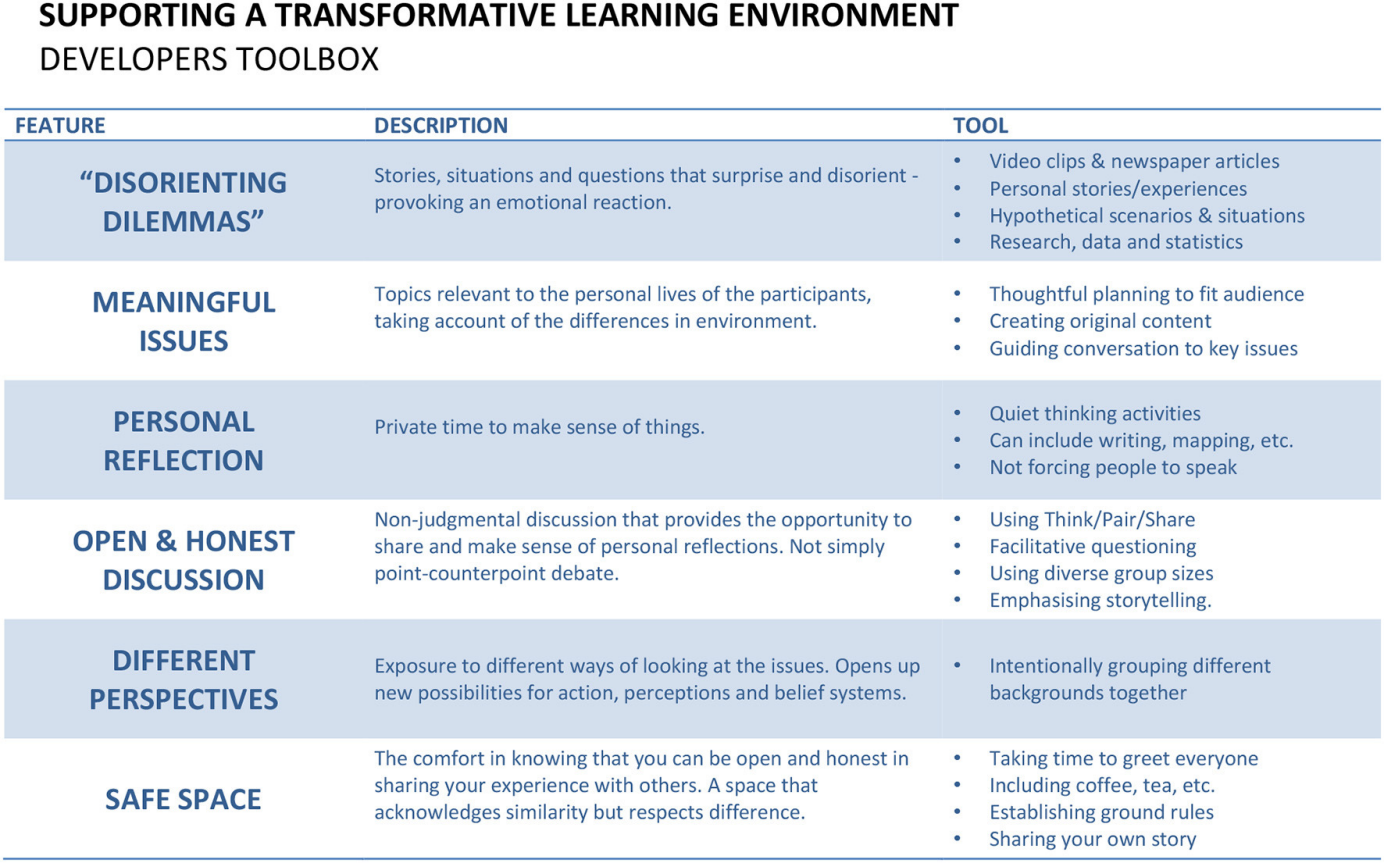
Transformative learning developer guide.

Centred on transformative learning and the Good Sports Spine, the team then developed a delivery framework that informed Good Sports community modules. Community sector professionals were identified and trained as facilitators (Good Sports developers) to deliver Good Sports community modules to parents, coaches, club, and school leaders in their own communities. A workshop delivery framework tool was also constructed to help developers follow the transformative learning framework (Figure 4).

The developer started each module with a powerful sport story (the disorienting dilemma). Clips from films such as *Trophy Kids* (Anderson et al., 2013), a documentary portraying four families' experiences for “an intense look at overbearing parents in sports” (IMDb, 2021), were included. Next, developers transitioned attendees into silent reflection followed by group discussions. Good Sports' delivery was flexible. Depending on time availability, and room, furniture, and group size, the developers' strategies ranged from writing silently, sharing with a partner, and small- and/or large-group discussions. Developers posed strategically designed questions, prompting authentic discussion related to the Good Sports Spine, including issues specific to attendees. A key aim of the discussion was leveraging the story and/or video so attendees reflected on their behaviours, attitudes, and assumptions. Developers provided handouts and slides, including the Good Sports Spine (Figure 3), to help attendees connect the story and/or video with their reflections before discussing. Sessions typically ended with the developer encouraging participants to share behaviour changes or intended future actions.

#### The good sports programme infrastructure

**Figure 5:**
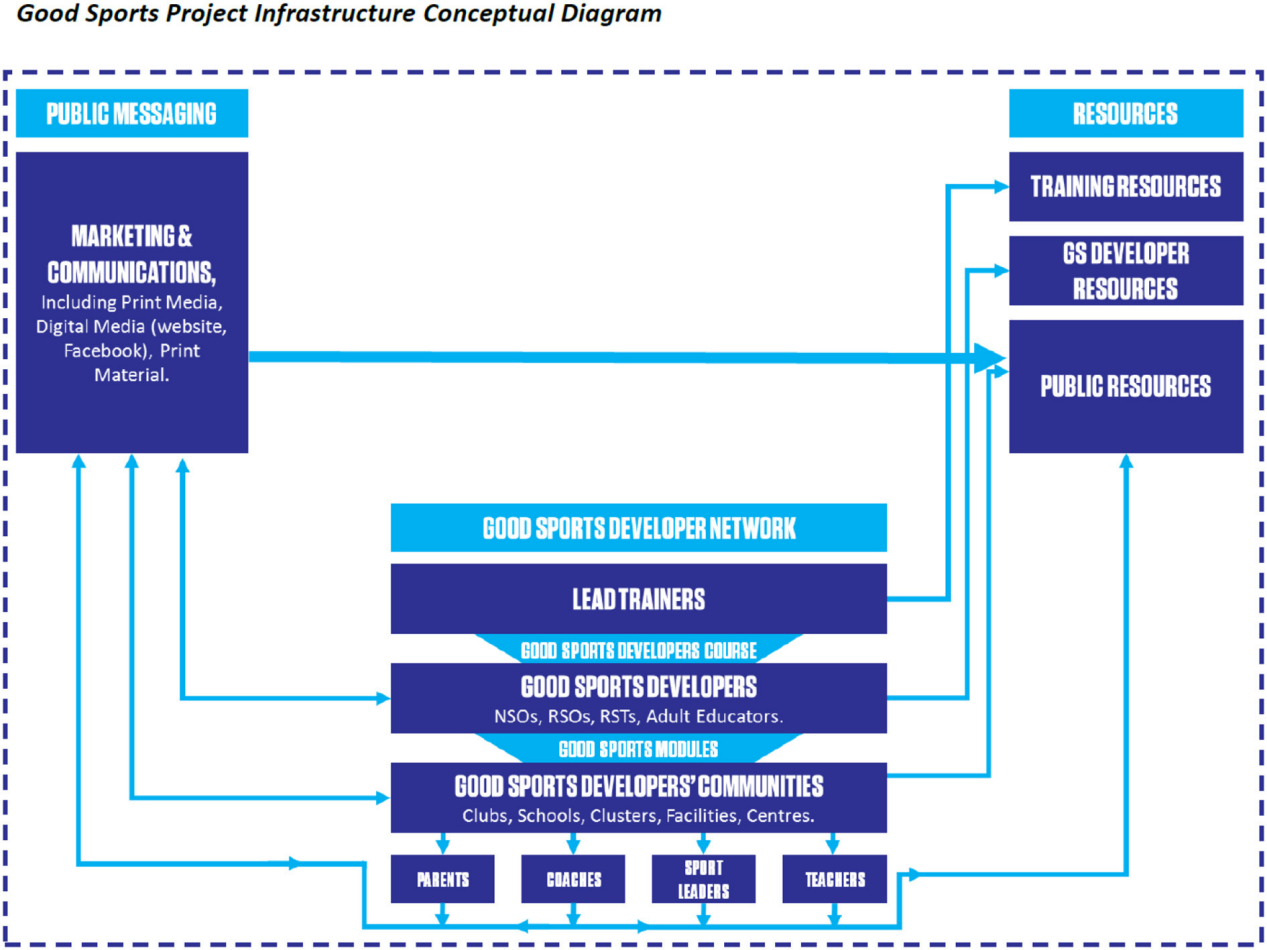
Good Sports programme infrastructure.

For effective rollout of community modules, the Good Sports team developed a three-pronged programme infrastructure (Figure 5): (1) The Good Sports developer network; (2) public messaging; and (3) resources.

The Good Sports' developer network reflects a “train-the-trainer” programme. Good Sports developers were supported within their own communities (clubs, schools, etc.) to deliver community workshops (typically 30–90 min) and receive ongoing professional development. To ensure quality delivery, lead trainers delivered a 2-day course to selected community sport professionals, who then became developers. Key areas addressed were: (1) research underpinning pertinent issues in junior and youth sport (i.e., the Good Sports Spine); (2) the community module delivery framework, including transformative learning principles. Aktive's marketing and communications team delivered public messaging, including on social media, news media, and websites, to raise awareness of Good Sports and key junior and youth sport issues.

#### Monitoring and evaluation

The first consideration for researchers adopting mixed methods research relates to purpose (Schoonenboom and Johnson, 2017). Common classifications of mixed methods' purpose have drawn upon the work of Greene (Greene et al., 1989; Greene, 2007), and include triangulation, complementarity, development, initiation and expansion. This classification has been expanded upon by subsequent researchers (e.g., Bryman, 2006), however the purpose of utilizing mixed methods in this project was that of “complementarity,” where different methods are used to explore, elaborate on, enhance and clarify different features of the same phenomenon (Schoonenboom and Johnson, 2017). The design of mixed methods' studies has tended to fall within two main groups: integrated (an interplay of methods throughout the study) and component (where each of the methods retains methodological separation) (Greene, 2007; Bazeley, 2010). The design adopted in this project reflected a component design, where methods remained discrete and independent throughout, and integration occurred as the findings were interpreted. Due to the iterative, non-linear nature of DBR, flexibility was important and some study results informed the design of subsequent studies, i.e., the approach was emergent rather than fixed. The research team designed methods to quantitatively “test” and measure, for example, changes in workshop participants' attitudes and beliefs. Qualitative methods were designed to explore and gain more nuanced and deeper understanding of different stakeholders' perspectives of the Good Sports programme, with no priority given to quantitative or qualitative approaches (QUAN + QUAL)

A key principle of DBR is that projects undergo multiple iterations. Implementation of the programme, initially designed to be over 2 years, extended to 3 years, as the project team and steering group recognised ongoing value in obtaining further feedback from diverse stakeholders. Sub-study design was therefore ongoing. Table 2 provides an overview of the methods utilised in each sub-study over the 3-year project.

**Table 2:** Methods.

**Data source**	**Description**	**Iteration**	**Participants**	**Aim(s)**
Narrative literature review	Inform survey design	Initial design phase	N/A	Inform the design and development of a survey measuring adults' attitudes towards children's sport participation.
Surveys	Community module survey (completed post module) Developer course survey (pre and post course)	Years 1 and 2 Years 1 and 2	246 adult (150 female, 96 male) coaches, parents, and club leaders. New Zealand or other European (58%), Māori (39%), Pacific Islander (19%), Asian (3%)^a^. 59 adult (38 male, 21 female) club and sport organisation leaders. New Zealand or other European (77%), Māori (7%), Pacific Islander (11%), other (12%).	Investigate the influence of community modules on adults' attitudes towards junior and youth sport. Establish: the workshop's effectiveness; how prepared the developers felt to deliver Good Sports workshops in their communities; any changes in thinking that occurred through participation in the workshops; developers' understanding of the transformative learning framework.
Case studies. Data collection methods included a mix of: Community modules Observation Field notes Semi-structured interviews Document analysis Focus groups Surveys	Developers' insights Primary school and family Parents (Master's study) Club administrators Inside researcher Industry-research nexus Developers' engagement Tertiary sport students Project manager Young people's experiences (Master's study)	Year 1 Year 1 Year 1 Year 1 Years 1-3 Years 1-3 Year 2 Year 2 Years 2-3 Year 3	One developer (female). Eight parents (5 female, 3 male), 12 teachers and Board of Trustee members (9 female, 3 male), one family (father, mother, 3 daughters aged 9, 11, and 13 years). 17 members (14 male, 3 female) of a volunteer junior rugby governance group. Two sport club administrators (1 male, 1 female). One adult male researcher (second author). Seven adults (5 male, 2 female) (4 industry partners and 3 academics). Eight adult developers (5 female, 3 male). Three tertiary sport students (2 male, 1 female). Good Sports project manager (male). 14 children (ages 7–13 years; 9 male, 5 female). Four parents (4 female) from a Pasifika community sports module.	Gain insight into the experiences of one developer who attended the first developer course through to her delivery of multiple community modules. Examine the influence of community modules on school boards, teachers, parents, and one family. Provide insight into the perspectives and experiences of parents who participated in a Good Sports community module. Provide insight into perspectives of the Good Sports programme; any changes implemented as a result of the programme; and thoughts related to Good Sports' future involvement at their club. Provide ongoing insight into the efficacy and evolution of the Good Sports education framework, resource development, and module delivery from a research and industry perspective. Examine the efficacy of a collaboration between a sport organisation and researchers; provide insights learned from key partners' perceptions. Examine developers' engagement levels with Good Sports' module delivery. Gain insight into the influence of a developer course delivered to tertiary sport students. Provide insight into challenges faced implementing Good Sports, and inform recommendations for a nationwide rollout of the programme. Capture the perspectives of children who participated in a ‘tag' rugby programme and parents who attended a Good Sports community module.

^a^Participants were given the option to identify with more than one ethnicity.

Participant recruitment varied depending on the nature of the sub-study. For developer modules, community sector professionals were identified by the Regional Sports Trusts and RSOs and trained as facilitators (Good Sports developers) to schedule and deliver Good Sports community modules to parents, coaches, club, and school leaders in their own communities. Acknowledging the complexity and challenges of conducting research in real-world settings, developers were afforded the flexibility to deliver modules based on “greatest need” or convenience. For case studies, purposive or snowball sampling was utilised to identify information-rich cases. For example, participants in the parents' case study were identified by a member of the research team as, potentially, representative of a “hard to reach” group. In another case study, a primary school was a desired setting and a school principal, who had heard about Good Sports and wanted to implement the programme, approached the research team.

The overall design of each sub-study was overseen by the lead author and inside researcher, in consultation with the project manager, industry lead trainer and steering group. This lead group also designed the developer and community module surveys, informed by the narrative literature review of survey design conducted in the design phase (see Table 2).

Community module surveys captured participants' demographic information, including role(s) played in children's sport. A five-point Likert scale was utilised to capture workshops' influence on participants' views regarding: what makes a positive sporting experience for young people; adults' roles in children's sporting experiences; and the purpose of sport for children. Each Likert scale question was followed by an open-ended question to gain deeper insight and a closing open-ended question to provide further information if required.

Developer surveys captured demographic information and asked participants the following open-ended questions pre- and post- the developer course. What makes a positive sporting experience for children? What makes a negative sporting experience for children? How would you define learning? What teaching or facilitating strategies do you believe promote learning? In the post-course survey participants were asked a five-point Likert scale question to establish their perceived level of preparedness to deliver a Good Sports community module.

For each case study, smaller teams were formed. Each team was led by one senior researcher and was responsible (with oversight from the lead team) for designing interview and focus group questions based on each study's aims (see Table 2).

#### The implementation phase

Throughout the implementation phase, the research team utilised a comprehensive monitoring and evaluation programme. Consistent with a DBR approach (Oh and Reeves, 2010), the monitoring and evaluation process informed the project's on-going design. There were three iterations over the 3-year pilot. Annual reports, based on the flexible mixed methods approach (Table 2), included findings from surveys, focus groups, semi-structured interviews, and multiple stakeholder-embedded case studies were informed by Stake's (1995), Baxter and Jack's (2008) work.

#### Analysis procedures

Focus groups and semi-structured interviews were audio recorded and transcribed verbatim. The research group was divided into smaller teams led by one senior researcher responsible for each sub-study. Descriptive statistics summarised quantitative data from closed survey questions. A six-step inductive thematic analysis was conducted to analyse data from semi-structured interviews, focus groups, and open-ended survey responses (Braun and Clarke, 2006). (1) Familiarisation with data occurred through transcription and initial note taking; (2) transcripts were read closely followed by generation of initial codes; (3) potential themes were discussed and named in group meetings; (4) themes were reviewed and agreed upon; (5) final themes were defined; and (6) findings were written. Teams met monthly to discuss the analysis as the studies progressed. Following a component design, sub-studies were analysed discretely and reports were produced. The final stage of our analysis brought together the findings of each sub-study and overarching themes were identified and presented in the form of overall annual reports (Bazeley, 2010).

In line with an emergent approach, more nuanced thematic analysis techniques were employed in the 2nd year of the project to add greater depth of understanding. For example, the young people's case study, conducted as a Master's study, adopted a more interpretive lens and used an SDT framework (Deci and Ryan, 2000) to inform data analysis (see Sadiman, 2017) for detail). A secondary deductive analysis of community module participants' open-ended survey responses in year 2 was further guided by Sterling's (2011) four levels of learning, examining changes in community module participants' perspectives and attitudes in greater depth. Analysis identified four distinguishable levels of influence ranging from surface-level change to deeper shifts in attitudes, questioning of assumptions, and suggested behavioural changes: Level one— “I already believe in”; Level two— “given me some good tips”; Level three— “certainly makes me think”; and Level four— “makes me re-evaluate my beliefs.”

## Results

Each data source/sub-study was treated as “one piece of the puzzle”, enabling greater understanding of the phenomenon studied (Baxter and Jack, 2008) and contributing to ongoing refinement of the developer course and community module delivery, and resource design, for example, the Good Sports Spine went through multiple iterations (see Figure 3 for the final version). During the implementation phase, the research group provided Sport NZ with annual, individual case study, and survey reports. Status reports were produced bi-annually for consideration and feedback from the steering group. That level of reporting detail is beyond the scope of this paper; however, Table 3 provides an overview of key findings from each sub-study.

**Table 3:** Key findings of good sports' sub-studies.

**Study**	**Key findings**
Community module survey	Attitude changes in coaches and parents. Resources resonate with participants. Disorienting dilemmas triggered emotional responses.
Developer course survey	Attitude changes in coaches and parents. Resources resonate with participants. Disorienting dilemmas triggered emotional responses. Positive impact on sector professionals.
Developer insights case study	Attitude changes in coaches and parents. Resources resonate with participants. Resource language needs to be appropriate for audience. Disorienting dilemmas triggered emotional responses. Positive impact on sector professionals.
Primary school and family case study	Attitude changes in teachers and parents. Disorienting dilemmas triggered emotional responses. Structural changes made to school sport practices following modules. Reinforced principal and parent attitudes - “good to see research backs up our beliefs.”
Parents case study	Attitude changes in parents. Resources resonate with participants. Positive influence on participants as “influencers” filter down to coaches and parents in a rugby setting. For greater detail see Ali (2018).
Club administrator case study	Attitude changes in coaches and parents. Positive impact on sector professionals. Structural changes made to club practices following modules.
Inside researcher case study	Attitude changes in administrators, coaches, and parents. Resources resonate with participants. Overcoming a perceived theory-practice divide. Disorienting dilemmas triggered emotional responses. Positive impact on sector professionals. Structural changes made following modules.
Industry-research nexus case study	Resources resonate with participants. Overcoming a perceived theory-practice divide. Collaboration success requires: grounded and practically based academics, industry to be open to the value of research, engagement of an insider researcher, and respect for all partners' contributions. For greater detail see Bradbury et al. (2021).
Developers' engagement case study	Resources resonate with participants. Positive impact on sector professionals. Level of developer engagement dependent upon employer's level of engagement with Good Sports.
Tertiary sport students case study	Resources resonate with participants. Disorienting dilemmas triggered emotional responses.
Project manager case study	Resources resonate with participants but more needed with specific New Zealand focus. Overcoming a perceived theory-practice divide. Structural changes made to organisation practices following modules. Developers need ongoing professional development and support. Develop a platform to disseminate research findings to a wider audience.
Young people case study	Reinforced national and international research in relation to what children value in sport. A child's ideal sporting environment should involve: freedom to socialise with friends and teammates; equal game time for all; opportunities to make their families proud; positive sideline comments to both teams; less emphasis on winning; and parents to show genuine interest in their sport participation. For more detail see Sadiman (IMDb, 2021).

In line with a DBR approach, the project manager, inside researcher, lead author, and industry lead trainer reviewed sub-study findings monthly to inform amendments to the developer course, community module delivery framework, and resources, and to ensure effective knowledge translation for the diverse participant groups. The lead author and project manager reported preliminary findings to the steering group, who identified further areas of potential inquiry as the project evolved. Annual end-of-year evaluation reports provided focus for discussions guiding the following year's approach and identified potential further areas of inquiry. As outlined in Table 3, Good Sports modules clearly influenced adult attitudes regarding junior and youth sport being more developmentally focused. Emergent case studies were designed to explore perspectives of a broader range of communities of interest.

### The final phase: Evaluation

On completion of the three-year pilot, the research group reviewed all sub-studies and produced a final evaluation report. Over the course of the pilot, 4,222 people attended developer or community module sessions. This section provides an overview of the evaluation's main findings.

#### High level themes

**Figure 6:**
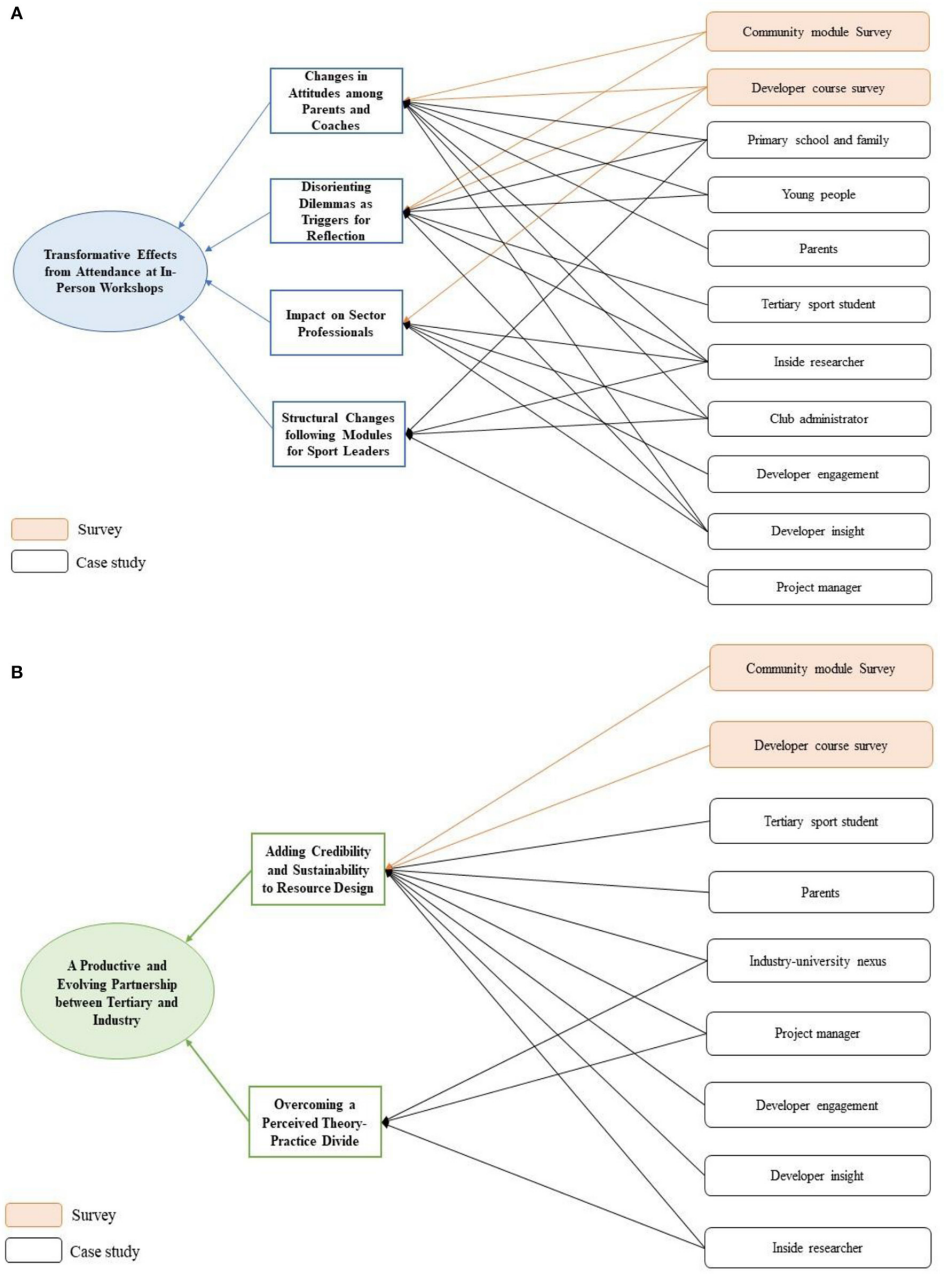
**(A)** Transformative effects. **(B)** A productive and evolving partnership.

Two overarching key themes were identified (see Figures 6A,B): (1) transformative effects from attendance at in-person workshops; (2) a productive and evolving partnership between tertiary and industry (see Bradbury et al., 2021).

### Transformative effects from attendance at in-person workshops

#### Changes in attitudes among parents and coaches

Survey responses revealed community modules positively influenced parents' and coaches' attitudes. Year one (96%) and year two (95%) respondents reported altered views on what comprises positive junior and youth sport experiences. Nearly two-thirds (63%) considered the workshop's influence very strong. Similarly, year one (86%) and year two (93%) respondents felt the module influenced their views on adult roles in junior and youth sport experiences.

Open-ended survey question responses revealed Good Sports community modules influenced perceptions of parents, coaches, and key adult influencers' roles to varying degrees. For those endorsing a “climate of development”, community workshops typically reinforced pre-existing views. Conversely, for those endorsing a “climate of performance”, community modules challenged their views, for example, “[previously] thinking I am helping my child with advice when I could be possibly putting her off the sport entirely” (Parent). These participants highlighted videos and discussions as key to prompting different attitudes. Some participants mentioned experiencing dramatic re-evaluation of their belief systems, including changing their strong belief in a “climate of performance”: “I usually have a car ride conversation with my 5-year-old [regarding game performance]. I now need to stop so my child can just have fun. I know my reaction, behaviour, etc., affects the way my child performs” (Coach). One parent shared some deeply self-reflective insights into the impact of a community module she attended:

I attended a Good Sports workshop for [my sport]…I am the parent that passes judgement sometimes, the one who picks at every little thing, the one that thinks they are helping but isn't, and I am so ashamed to admit that. I thought I was giving her helpful advice and leading her down the path to success, and although those were my intentions, the way I was getting that message across was just wrong. I left that workshop feeling so heartbroken for my poor baby, who is only 10 years old, and always tries her damn hardest at everything she does. Yet, here I was ruining these special moments for her by being a sergeant, as opposed to just being her mum. I got home and was telling my partner about the workshop and I just broke down in tears. I don't want to be THAT parent anymore. I then went and asked my princess [daughter] what her favourite thing about [her sport] was and she replied “I love being with my friends, mum, and learning new skills.” I hugged her, told her I loved her, and promised I wasn't going to be THAT parent anymore. If you ever get a chance to attend a workshop like this, please go. Your whole perspective on this subject will change for the better of not only you, but your children too (Parent).

Survey responses produced very little negative feedback. Specifically, <2% of participants' responses were critical of Good Sports, the majority of which were regarding the workshop's structure. For example, “I am not sure your connections in the session flowed, so the Good Sports stuff warranted more mediation before you fired off the discussion questions” (Club leader).

Case studies examined parents' experiences of a community module. Pre- and post-module interviews with a single family revealed how the course reinforced their views of junior and youth sport and, specifically, parents' roles. Prior to the module, the parents endorsed open, supportive approaches. They volunteered for “parent duties” at sporting events, were concerned about abusive sideline behaviour, and resisted pressuring children. Following the community module, the father continued to endorse these views but felt strongly that “the ones [parents] who were missing were the ones who should have been there [at the module].”

Survey data suggested community modules heightened coaches' and parents' awareness of negative behaviours and initiated changes in thinking. One representative-level junior coach mentioned the module affected his approach towards parents; consequently, he encouraged them to contribute positively:

Last year I put a real strong emphasis on the parents to stay out of it [not providing input related to trainings and games], [be]'cause it [is] such a pressure pot of a situation. This year I'm putting more emphasis on everything that the parents say has got to be positive.

#### Disorienting dilemmas as triggers for parent reflection

Survey comments and case study findings reinforced transformative learning's effectiveness as a community module framework. One developer noted the importance of effective disorienting dilemmas:

The parents really did get a lot from the car ride home [video]. It's not to that extent [of the behaviour in the video] but they do actually talk to the kids on the way home about what they could have done better [in the game], and they realised actually how damaging that could be with their kids maybe wanting to quit the sport because of those conversations. That's been really powerful, the parents watching that video.

The developer's comment suggests Good Sports community modules highlight taken-for-granted views that undermine positive junior and youth sport experiences, which some parents and coaches reinforced: “It has shown that parents' influence is more important than we think” (Parent). “I do want to find out from my boys what they enjoy about playing” (Parent). “A lot of my interaction has been what they can improve on, without asking what it is they enjoy. [The module] changed my view on how to coach this age group” (Coach).

Furthermore, the IR described how emotional connections were established at community modules:

An essential element to the Good Sports process has been the regular use of “disorienting dilemmas” to provoke an emotional response in participants. Employed initially by scenes of overbearing parents in the American documentary *Trophy Kids*, powerful stories and situations generate reactions in participants that lead naturally into conversation.

Some parents acknowledged the value of working through complex issues with peers in mutually supportive environments: “Great opportunity to come together with other parents,” and “I liked the idea of practising and working in a mutual participation and development zone.”

#### Impact on sector professionals

Originally, parents and coaches were the target of Good Sports. However, as the project evolved, the need to influence regional and local sport leaders became apparent. Case study and survey data indicate developer courses influenced and reinforced sector professionals' attitudes and beliefs towards strongly supporting a “climate of development.” Some developers overcame initial reticence by viewing the videos, reflecting on their attitudes, and discussing issues. According to the IR,

There are people who come to the developer course who go through their own cathartic experience. We get some tears there. We get people who are initially resistant but then they come around. And I think that says something really important.

A common theme from the surveys was developer courses were rewarding, eye-opening experiences that fostered emotional connection to junior and youth sport issues: “I really, really enjoyed this workshop, thank you. I walked in with my own perceptions and some have been changed. [The workshop] challenged my way of thinking, and I want to be a change agent!” (Developer). The developer course surveys revealed that many participants felt positively about the topics and approach taken. For example, “Interesting, loved listening to all the diversity of thought and experience in the group.”

In this way the Developer course unexpectedly became a form of professional development for the sector. Acknowledging this development, The IR believed “carefully facilitated discussions using targeted content and support resources underpinned by research” focused attention on developers' underlying beliefs and assumptions. The IR also felt implementing transformative learning principles enabled deeper levels of learning, noting it was “a framework that embraces powerful stories, emotional issues, meaningful conversation, and a diversity of perspectives.” Consequently, the Developer course revealed gaps in understanding among Developers, followed by a wave of interest in filling such gaps.

While many Developers had positive experiences with the programme, not all became fully engaged with the programme's aims. In the early stage of the project, it became clear that there were three types of Developers: (1) The “enthusiasts,” who the IR described as “a group of developers who are very enthusiastic, jump into it right away, want to get better and run more workshops”; the “samplers,” “who run one or two then kind of stop”; and (3) the ‘indifferent,' “who aren't doing anything.” Importantly, inconsistent forms of engagement may have been the result of limited support from the project team for Developers after attending the Developers course, who may need more time to engage with and reflect upon the issues. In fact, the IR and project manager originally felt that the Developer Course should last multiple weeks, but this was deemed impractical given the limits on these professionals' time and work lives. For the IR, the Developers' experiences with learning and delivery highlighted the business of sector professionals and speaks to the challenge and complexity of achieving culture change in youth sports.

#### Structural changes following modules for sport leaders

Delivery of modules to club-, regional-, and national-level organisation leaders resulted in positive effects across the sector. Specifically, the administrators' case study revealed Good Sports sparked change in a rugby community where young people traditionally faced high expectations, including pressure to perform, specialise early, and turn professional. The rugby club president noted too much previous focus on winning. The administrator stated that, consequently, the club committed itself to instituting multiple Good Sports community modules annually: “Our [president] and [club manager] are committed to ensuring every parent, coach, and adult influencer attends a community module next year, prior to the season.”

Similar experiences occurred within some Regional Sport Organisations (RSOs) and National Sport Organisations (NSOs). A bespoke Good Sports 2-day module was delivered to one NSO's Regional Development Officers; subsequently, Good Sports was integrated into their nationwide coach development process. Two RSOs included Good Sports modules in their programme structure and coach education, with the Good Sports Spine integrated into their coaching material. In response to declining junior and youth participation, higher injury rates, and inadequate developmental pathways, North Harbour Rugby Union (NHRU) discontinued its representative junior teams, which was endorsed by the national governing body, New Zealand Rugby. Significant social media backlash, and national and international media attention resulted (Schofield, 2019), focussing on reasons and research underpinning the move. Other provinces and codes followed suit. Good Sports was not the sole trigger for RSOs' actions; however, conversations with the Good Sports team reinforced organisations' beliefs their approaches were appropriate and provided research supporting their decisions.

Good Sports' impact on RSOs and NSOs highlights the programme's potential to catalyse structural change in New Zealand junior and youth sport. One industry-academic nexus case study participant described the situation as:

A perfect storm. At the onset of Good Sports, we were not overly optimistic about the influence that Good Sports could have on influencing a societal shift in thinking. We felt we could develop a programme that would influence adult-coach and parent thinking. Influencing policy change was something we thought was possibly an unrealistic aim in the current climate. However, the combination of factors and issues that have focused societal attention on youth sport have created a space where people are interested and want to hear about the research, and many organisations are ready for change.

Importantly, the IR issued a word of caution regarding the long-term sustainability of Good Sports as a culture change initiative. Specifically, genuine concerns exist that Good Sports would be narrowly understood as simply a workshop that might become a checkbox item for sector leaders. In that sense, if sector leaders do not authentically understand the spirit of Good Sports, including the Climate of Development as a youth sports philosophy (Galatti et al., 2016; Bean et al., 2019), then the project may not ultimately influence the structural constraints that perpetuate a Climate of Performance but remain invisible to leaders. In complex environments, patterns of behaviour emerge from the interaction between elements of a system (Meadows, 2008; Sen, 2012). As an example, it became clear to many within the project that parent and coach behaviours could not be acted on without simultaneous engagement with sector structures and policies, such as season boundaries, training-to-competition ratios and competition models. Acknowledging this “elephant in the room,” the IR noted his long-term desire to see Good Sports spark internal reflections between Developers and their organisations about the ways in which they structure their sport and the implicit messages that those structures communicate to coaches and parents:

we have trained them to be developers and they have really emotionally bought into these issues. If they go back and have a conversation with their superior or their organization about this I see that as part of the culture change process, but the fact of the matter is that the “elephant in the room” for this programme is how do we engage the *structural constraints* that have a negative impact on kids' experiences, that in fact may be promoting that performance climate more so than the belief system of the parents.

### A productive and evolving partnership between tertiary and industry

Industry-research nexus case study participants believed the industry practitioners and academics' relationship enhanced Good Sports' effectiveness. The group collaborated throughout the study, initially reviewing and refining the delivery style, establishing developer understanding of the adult learning framework that underpinned module delivery, and continually refining and adding to project resources (see Bradbury et al., 2021). Two elements in this process warrant specific attention: (1) adding credibility and sustainability to resource design and (2) overcoming a perceived theory-practice divide.

#### Adding credibility and sustainability to resource design

Resources supporting Good Sports' delivery framework were designed collaboratively and based on current junior and youth sport research. A central illustration is the Good Sports Spine (Figure 3). The industry-academic (research) nexus case study revealed the Good Sports Spine's design and implementation was critical for participants, facilitators, and project stakeholders. For developers, this tool added credibility: “When you're dealing with parents, particularly parents that don't want to believe in what you're doing, giving them stats and research reiterates what it is you're trying to deliver.” At the project level, the project manager and lead trainers viewed the spine as a tool that resonated across codes, genders, communities, and competition levels.

The spine has become synonymous with Good Sports and gained national recognition. I imagine it will still evolve, but I think we've got some life span with where it is at the moment. It's a significant artefact, and the feedback has been pretty good to date around helping people on the ground navigate all those complex issues (Project Manager).

#### Overcoming a perceived theory-practice divide

Interviews with lead trainers and research group members revealed the project overcame a perceived historical divide between research and practice in New Zealand sport. First, the integration of a lead trainer (the IR) with both academic and practical coaching and pedagogy expertise added considerable value. The IR drove academic dimensions of the adult education delivery and format, drawing upon a transformative learning framework. One industry lead trainer said the following about the IR:

He lived and breathed the stuff [academia, and sport coaching and pedagogy], which added in-depth insights and a research base. Having him as an academic researcher was important. At the end of the day, a framework that he had created had to be delivered in an understandable way. I think that was the real art of this project.

The second lead trainer and project manager felt comfortable challenging academic concepts with the IR and the research group leader, including language and delivery that created confusion or failed to resonate with coaches, parents, and key stakeholders' experiences. For instance, the spine evolved through an iterative process that included project manager feedback, the research group producing resources, and experimentation and piloting in a community setting.

That said, the work of bridging theory and practice demanded intentional boundary crossing and reflexivity. Consistent with current understandings of insider research (Wiser, 2018), the IR faced an ongoing challenge to negotiate tensions in their dual position as lead trainer and researcher. For instance, the IR was employed as a part-time contractor with pre-identified research expertise in theories of learning. During the early phases of the project's design, firm boundaries were drawn between the IR's focus on adult learning theory and the industry team's focus on sport content, including current issues in sport coaching. Given the IR's extensive experience as a sport coach, this positioning challenged their identity as a practitioner while also reinforcing their outsider status as an academic. Such tensions prompted critical reflection around how to engage the industry team:

In terms of roles we had developed a sort of recognition I was coming more from the research perspective and [industry lead] was coming from a practitioner perspective. I don't 100% agree with the dichotomy as I view myself as a practitioner as well. But for the purposes for the project it worked pretty well. And I think that I was always managing how far do I go, how far do I push, as I am a contractor so literally an outsider. How far do I push in terms of these areas that I see as potentially gaps that could affect the success of the programme that aren't my explicit role?

Conflicts associated with researcher identity and insider-outsider status highlight the importance of reflexivity among researchers engaged in community and/or design-based research in sport, especially inside researchers (Greene, 2014). An academic and lead trainer described this as “living in intersecting multiple worlds” and one industry lead trainer viewed it as essential to the project's success.

One research group member hoped the “perception that academics were a little removed from the “real world” was broken down and was no longer considered a major barrier.” However, they supported the view that academics working in such environments “need to be a good fit” and, ideally, practitioners “on the ground.” The research group comprised coaches, athletes, and managers. Importantly, the need for mutual understanding was reciprocal. From an industry perspective, a lead trainer noted “industry people need to be open-minded” and “cognisant of the latest junior and youth sport research.” In this respect, Good Sports benefitted from Aktive staff, including lead trainers and the project manager, who were experienced and skilled workshop facilitators involved in sport for many years at various levels.

#### Recommendations

Four key recommendations were made in the final evaluation report.

(1) Good Sports becomes a national programme. At its core, the issues that Good Sports addresses speak to broader concerns for New Zealand. At a time of concern regarding individuals and communities' wellbeing, and the perceived “professionalization” of school sport, Good Sports represents an opportunity for Sport NZ to lead the way internationally promoting culture change in junior and youth sport.

(2) Development of more specific New Zealand-based content. The success of *Trophy Kids*, an American documentary, in a New Zealand programme signifies the power of emotionally provocative stories to generate discussion. Creating New Zealand-specific videos, articles, and research will, potentially, enhance this. Sport organisations and programme leaders are strongly encouraged to create genuine New Zealand stories that reflect the most relevant New Zealand sport issues. Further, a more intentional effort to integrate New Zealand sport-related research by New Zealand academics is encouraged.

(3) Lead trainer development. Lead Trainers shepherd Good Sports. For this reason, the identification and ongoing support of Lead Trainers is integral for Good Sports' effectiveness. Lead Trainers require more in-depth understanding of key issues in junior and youth sport and the dynamics of transformative learning.

(4) Expanding the research group's role. Considering the broader role of a “research group” would be beneficial to other sports projects or issues. As evidenced in this project, research groups can contribute to the design and execution of community-based initiatives. The positive relationship between industry and tertiary partners in this project can be perceived as an example of “best practice” in this regard.

## Discussion

Unlike recent international efforts to address problematic adult behaviour in junior and youth sport nationally, this study presents findings from a novel pedagogical approach. Design-based research was utilised to inform Good Sports' design, implementation, and ongoing evaluation. Findings deemed the collaborative and iterative approach essential to both the project and research group's aims. Specifically, to accurately interpret the findings, eight facets of DBR warrant attention (Oh and Reeves, 2010).

(1) Being situated in real educational contexts: The design of developer and community modules drew heavily from adult learning principles and, specifically, transformative learning. Adult views on the role and purpose of children's sport can be deeply entrenched (Walters et al., 2011); therefore, designing powerful triggers promoting critical adult self-reflection was important. The *Trophy Kids* video (IMDb, 2021) provided “disorienting dilemmas” (Mezirow, 2011), which were catalysts for module participants' reflections. The modules aimed to encourage re-evaluation of adults' beliefs and commitment to behaviour changes that reflect this (in line with Sterling's (Sterling, 2011) level-four change in relation to transformational learning). For example, one community module participant stated, “It makes me never want to be like those [adults] in the video. I will explain what I saw to others - I will share it. Kids really do get affected by it [adults endorsing a climate of performance].”

(2) Designing and testing a significant intervention to address complex issues: Significant groundwork was laid during the initial design phases and revealed that many countries were, mostly unsuccessfully, grappling with strategies to try to address junior and youth sport issues. A key focus was ongoing evaluation to monitor and regularly review the intervention's progress and efficacy.

(3) Using mixed methods: Regular steering group, project team, and research group discussions determined a broad range of data collection methods to capture multiple stakeholders' perspectives.

(4) Including multiple iterations: Three significant implementation-phase iterations were included. The research team produced annual reports drawing from all data sources, which were discussed and reviewed by the steering group. Aktive staff and researchers used findings to continually refine and test key aspects of the intervention.

(5) Incorporating a collaborative partnership between researchers and practitioners: This was evident through an ongoing industry-research nexus case study reviewing the relationship's efficacy and detailing its successes and challenges to Sport NZ. This study, based on (Mayer and Kenter, 2015) collaboration theory framework, provided guidelines for academics and industry to work together more effectively in applied sport settings (Bradbury et al., 2021). Three recommendations, identified by Mayer and Kenter as important determinants of successful collaborations, were crucial to building social capital and trust. First, academics engaging with industry in applied sport settings need to ideally be practitioners (coaches or managers) and “grounded.” Second, industry partners need to be open to, and acknowledge, the value of research to projects. Third, mutual respect is fundamentally important to the relationship's success.

(6) Evolution of design principles: This occurred at each iteration's conclusion. Drawing upon findings from data sources, refinements to the delivery framework, resources, and media messaging followed each iteration at the end of years one and two.

(7) Comparison to action research: This study's DBR approach clearly overlaps with action research where continuous improvement towards research outcomes is sought through collaborative work influencing ongoing modifications of iterations (McNiff, 2013). The steering group, researchers, and Aktive held regular meetings, and the principle of co-design, continuous reflection, and refinement were key elements of Good Sports.

(8) Practical impact on practice: Impact on practice was examined by capturing multiple stakeholders' perspectives and evidenced by policy and structural changes that sport organisations who participated in Good Sports introduced. This included embedding Good Sports modules in national and local frameworks, replacing representative sport competition structures with developmental programmes aligned with a “climate of development,” and, at an individual level, coaches and parents who attended community modules committing to behavioural changes.

This study's findings suggest programmes underpinned by research and robust pedagogy that include face-to-face interactions such as the MAC programme (Topor and Gill, 2008) can influence adult attitudes and belief systems. Negative feedback was negligible, and those whose perspectives remained unchanged following Good Sports modules predominantly stated they “already thought this way.” Importantly, the effectiveness of the module delivery framework based on principles of transformative learning (Mezirow, 2011; Taylor, 2011) exceeded the project group's expectations. The module's disorienting dilemma prompted discussions between attendees and videos such as *Trophy Kids* provided powerful scenes that generated emotional connections necessary for re-evaluating problematic beliefs.

Findings reinforce the need to consider structural and environmental constraints within projects aiming to initiate adult behaviour change. Increasing evidence suggests the nature of parents' involvement in junior and youth sport is not always within their control/volition. For instance, evidence from the United States and United Kingdom indicates sport culture, expectations, and norms impact parents' behaviour (Clarke and Harwood, 2014; Dorsch et al., 2016, 2019). Similarly, organisations and parents' expectations influence coaches' behaviour (Walters et al., 2012). Early signs indicate that Good Sports has positively influenced some noteworthy structural changes in New Zealand, such as NHRU, through targeted advocacy and education for club-, regional-, and national-level sport leaders.

The project's intent was to initiate culture change regarding how junior and youth sport is perceived in New Zealand. Recent New Zealand events support this movement. Johanssen (2020), acknowledging Good Sports' role, described a “seismic shift” in junior and youth sport in New Zealand. In 2019, junior and youth sport structural and policy changes were introduced in New Zealand. CEOs from five leading New Zealand sports (rugby union, football, netball, cricket, and hockey) signed a Statement of Intent to implement changes advocating a “climate of development” in junior and youth sport (Sport NZ, 2019b). In recent months, 10 further NSOs also signed. Furthermore, a number of sports made changes to delay representative competitions until participants were at least 15 years of age (Bidwell, 2021).

It is difficult to measure Good Sports' influence on broader cultural shift of thinking in New Zealand. Good Sports provided information and theory from recent junior and youth sport research, which organisations utilised to justify structural and policy changes. The Good Sports project worked closely with several sport organisations, and deeper understanding of junior and youth sport research resulted in shifting social junior and youth sport discourses. However, as we write, the death of a young elite New Zealand cyclist and allegations of psychological and verbal abuse, body shaming and sexualisation of young gymnasts have fronted “sport news” (Zydney et al., 2020; George, 2021). Winning the hearts and minds of parents and those involved in grassroots-level sport is only part of the solution. As Bidwell noted, a system change is needed and those who implement policy and design competitive structures need to be accountable. The evaluation of Good Sports suggests attitudes and beliefs can be positively influenced, but more is needed at all levels for New Zealand to fully embrace a junior and youth sport system focused on wellbeing and enjoyment, rather than performance.

It has been suggested that the longer-range aim of DBR studies is to create findings generalisable through “multiple cycles across various settings” (Zydney et al., 2020, p. 12), however, we make no claim that DBR studies such as this are generalisable. Similar to case studies, this study is context specific, but we concur with Stake (Kidman et al., 1999) that contextual studies offer opportunities for ‘naturalistic generalisation' whereby readers recognising similarities between their research and experience can inform their own practice with these findings. This study was set in New Zealand, a country with close to five million people where personnel from various sport organisations often know each other. Delivering face-to-face frameworks in such projects would likely be harder in more densely populated countries. However, as evidenced in this study, face-to-face interactions and conversations in environments drawing from adult learning principles are highly valuable. We highly recommend that sporting organisations grappling with similar issues in their countries consider funding workshop approaches. We acknowledge that this can be an expensive approach to consider at a national level, but we argue that the potential benefits to young people outweigh the financial costs. More longitudinal studies drawing upon young people's voices are needed in New Zealand to evaluate whether the influence on adult attitudes and behaviour generated by this project ultimately results in enhanced experiences for young people.

Ultimately, the scalability of train-the-trainer, workshop-based models may depend on the degree of institutional collaboration across sectors, particularly funders, researchers and providers. The research team's experience negotiating tensions between academia and industry speaks to the challenges of collaborative design initiatives in sport and educational settings, including the need to build trusting relationships and a shared vision. When conducting community-based research in sport, Whitley et al. (2014) have noted that researchers must negotiate issues of trust, power and cultural competency. Such matters were certainly evident in this design-based research and cannot be taken lightly.

## Conclusion

The Good Sports project arose from shared concern between New Zealand government agencies and researchers regarding junior and youth sport experiences, gaining significant media attention in New Zealand and abroad. Good Sports was perceived to positively influence adult thinking at levels ranging from national government agencies and NSOs to community clubs, schools, and families. Following a 3-year pilot in Auckland, a nationwide rollout of Good Sports was piloted, and Aktive, subsequently, worked with organisations throughout New Zealand embedding Good Sports in their structures. In 2021, Sport NZ confirmed Good Sports national rollout, providing basis for its “parent education” approach.

A key finding from this study is collaboration between industry practitioners and academics is more likely to lead to meaningful and impactful solutions. Furthermore, meaningful face-to-face conversations to influence and change people's thinking, attitudes, and beliefs are recommended to others considering such projects. The final words belong to a developer from the Pasifika community:

I have always believed my role was to develop our people, I just didn't have the framework or words to explain it. Good Sports has provided both of those. I am really excited about the conversations I will now have with my community.

## Data availability statement

The raw data supporting the conclusions of this article will be made available by the authors, without undue reservation.

## Ethics statement

The studies involving human participants were reviewed and approved by AUT Ethics Committee (AUTEC), Auckland University of Technology. Written informed consent to participate in this study was provided by the participants and/or participants' legal guardian/caregiver.

## Author contributions

SW, VM, TB, and SS contributed to conception and design and implementation of the study. SW, VM, TB, PL, and KS conducted the analysis of the sub-studies. SW and VM conducted final overall evaluation analysis. SW wrote first draft of manuscript. VM and AL wrote sections of the manuscript. All authors contributed to manuscript revision, read, and approved the final submission.

## Funding

The overall project was funded by Sport NZ, awarded to Aktive Auckland (a charitable trust) who implemented the project. The research team was involved in the co-design and led the evaluation of the project but received no funding. Two Masters student scholarships of $14,000 each were embedded within the project and were funded by Aktive.

## Conflict of interest

The authors declare that the research was conducted in the absence of any commercial or financial relationships that could be construed as a potential conflict of interest.

## Publisher's note

All claims expressed in this article are solely those of the authors and do not necessarily represent those of their affiliated organizations, or those of the publisher, the editors and the reviewers. Any product that may be evaluated in this article, or claim that may be made by its manufacturer, is not guaranteed or endorsed by the publisher.

## Footnotes

^1^ACC is a government-funded organisation that offers financial assistance for treatment and compensation to all New Zealanders who sustain an accidental injury.

^2^Aktive is a government-funded organisation that “provides region-wide leadership, strategic direction, and a single point of contact for all sport and recreation stakeholders operating across Auckland” (Sport NZ, 2019a).
